# Metabolic neuroimaging of myalgic encephalomyelitis/chronic fatigue syndrome and Long-COVID

**DOI:** 10.1097/IN9.0000000000000068

**Published:** 2025-09-12

**Authors:** Yijuan Zhu, Patrick Quan, Tadahiro Yamazaki, Anna Norweg, Benjamin Natelson, Xiang Xu

**Affiliations:** 1 BioMedical Engineering and Imaging Institute, Icahn School of Medicine at Mount Sinai, New York, NY, USA; 2 Department of Neurology, Icahn School of Medicine at Mount Sinai, New York, NY, USA

**Keywords:** neuroimaging, metabolites, metabolic imaging, magnetic resonance spectroscopy, positron emission tomography, ME/CFS, Long-COVID

## Abstract

Myalgic Encephalomyelitis/Chronic Fatigue Syndrome (ME/CFS) and Long-COVID are complex, disabling conditions that have emerged as significant public health challenges, affecting millions worldwide. Despite their growing prevalence, effective diagnostics and treatments remain limited, largely due to an incomplete understanding of their underlying pathophysiology. Both conditions share hallmark symptoms of chronic fatigue, cognitive dysfunction, and postexertional malaise, but their biological underpinnings remain to be elucidated. Neuroimaging offers a promising, noninvasive window into the brain’s metabolic landscape and has the potential to uncover objective biomarkers for these conditions. In this mini review, we highlight recent advancements in metabolic neuroimaging, particularly positron emission tomography and magnetic resonance imaging/magnetic resonance spectroscopy, that reveal alterations in glucose and oxygen metabolism, neurotransmitter balance, and oxidative stress. These insights point toward shared disruptions in brain energy metabolism and neuroinflammatory processes, which may underlie the persistent symptoms in both ME/CFS and Long-COVID. Importantly, while some findings overlap, inconsistencies in metabolite profiles between ME/CFS and Long-COVID underscore the need for further stratification and longitudinal research. Standardizing definitions, such as identifying Long-COVID patients who meet ME/CFS diagnostic criteria, could help improve study comparability. By summarizing current imaging evidence, this review underscores the potential of neuroimaging to identify imaging biomarkers to advance the clinical diagnosis of Long-COVID and identify therapeutic targets for treatment development. As we continue to face the growing burden of Long-COVID and ME/CFS, metabolic imaging may serve as a powerful tool to bridge gaps in knowledge and accelerate progress toward effective care.

## 1. Introduction

**Figure 1.**
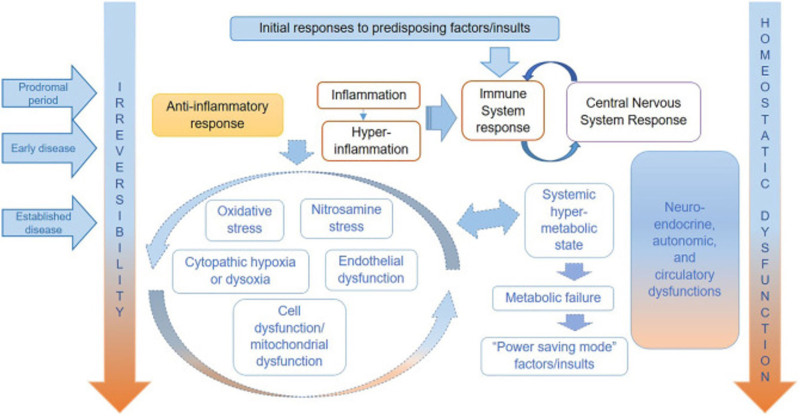
**Hypothesized pathophysiological mechanisms for ME/CFS, as summarized by Nacul et al ^[4]^**. Initially marked by neuro-immune hypermetabolism resulting from early inflammatory and immune-central nervous system responses, the disease evolves into multi-system dysfunction and a hypometabolic, low-energy state. As the disease progresses, irreversibility and homeostatic dysfunction increase. Reproduced from *Front Neurol*, 2020. 11: p. 826. ME/CFS, Myalgic Encephalomyelitis/Chronic Fatigue Syndrome.

Myalgic Encephalomyelitis/Chronic Fatigue Syndrome (ME/CFS) is a debilitating disorder characterized by profound fatigue that is not alleviated by rest, along with other symptoms such as postexertional malaise, unrefreshing sleep, orthostatic intolerance, and cognitive dysfunction ^[1,2]^. These symptoms significantly impair daily functioning and affect up to 17–24 million people worldwide ^[3]^. Despite being recognized as a neurological disease for decades, the underlying mechanisms of ME/CFS remain elusive, and there is a considerable gap in understanding its pathophysiology. The hypothesized pathophysiological mechanisms for ME/CFS, as summarized by Nacul et al ^[4]^ is shown in Figure 1. A wide range of viral infections, including coronaviruses, have been known to trigger postviral fatigue syndromes and subsequent ME/CFS ^[5–8]^. For instance, after the 2003 severe acute respiratory syndrome (SARS) epidemic, many survivors experienced prolonged health issues, with 60% reporting persistent fatigue and 44% experiencing sleep disturbances 6 months postinfection ^[9]^. A 4-year follow-up study of 233 SARS survivors found that 40.3% reported chronic fatigue, and 27% met the 1994 Centers for Disease Control and Prevention (CDC) criteria for ME/CFS ^[10]^.

**Figure 2.**
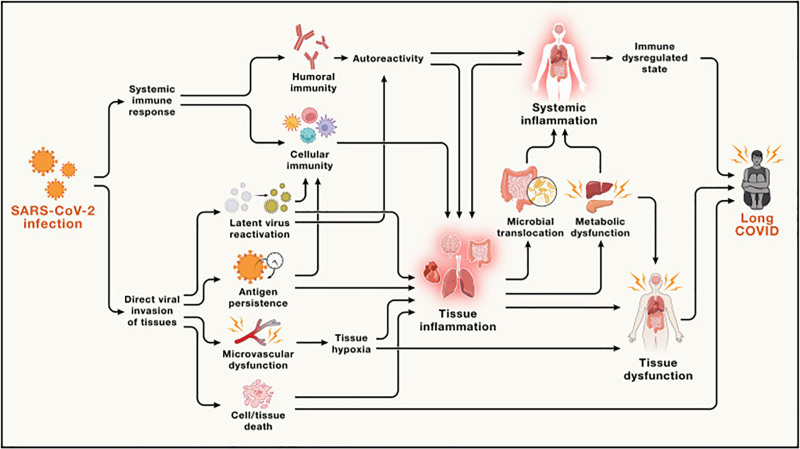
**Hypothesized mechanisms of Long-COVID, adapted from Peluso and Deeks ^[14]^**. Acute SARS-CoV-2 infection can trigger immune dysregulation, viral persistence, and tissue injury, leading to systemic inflammation and multi-organ dysfunction, particularly affecting the brain. Feedback loops between immune and metabolic dysfunction are thought to sustain chronic symptoms. Reproduced from *Cell*, 2024, 187(20): 5500–5529.

Following the recent COVID-19 pandemic, a large and increasing number of patients who were infected with severe acute respiratory syndrome coronavirus 2 (SARS-CoV-2) continue to experience a constellation of symptoms, including fatigue, dyspnea, autonomic dysfunction, and cognitive impairment, long past the time of initial illness ^[11–15]^. These symptoms, ranging from mild to incapacitating ^[16]^, are collectively referred to as Long-COVID or Post-Acute Sequelae of SARS-CoV-2 infection (PASC). During the first few years of the pandemic, PASC or Long-COVID was loosely defined; however, the World Health Organization (WHO) now defines Long-COVID as the continuation or development of new symptoms 3 months after the initial infection, persisting for at least 2 months without an alternative explanation ^[17]^. This definition underscores the chronic nature of Long-COVID, which closely resembles ME/CFS. Estimates suggest a conservative incidence rate of 10% of Long-COVID among those infected with SARS-CoV-2, potentially affecting at least 65 million individuals worldwide ^[18–22]^. Recently, a new study of COVID survivors remaining ill for at least 6 months noted 4.5% of patients fulfilled criteria for ME/CFS ^[23]^. The hypothesized pathophysiological mechanisms for Long-COVID, as summarized by Peluso and Deeks ^[14]^ is shown in Figure 2. Similar to ME/CFS, mitochondrial dysfunction, redox state imbalance, impaired energy metabolism, and chronic immune dysregulation are likely to be the main hallmarks of Long-COVID ^[22,24]^.

Compared with Long-COVID, ME/CFS is highly heterogeneous, with considerable variation in disease triggers ^[4,25]^, duration ^[26]^, symptom severity ^[27,28]^, and treatment response. This heterogeneity complicates efforts to pinpoint a singular cause or mechanism underlying the disease. Hypothetically, Long-COVID represents a relatively less heterogeneous group; all patients develop symptoms following SARS-CoV-2 infection ^[29,30]^, have a shorter disease duration, and are often unmedicated ^[31,32]^. Studying the physiological alterations in Long-COVID may therefore offer insights into the mechanisms underlying ME/CFS ^[33–37]^.

Neuroimaging techniques, such as magnetic resonance imaging (MRI) and positron emission tomography (PET), have been used extensively to study neurological diseases such as multiple sclerosis ^[38]^, epilepsy ^[39]^, Alzheimer’s disease ^[40]^, and psychiatric disorders ^[41]^. Even though ME/CFS has been recognized as a neurological disease for a few decades now, the neuroimaging features of ME/CFS are still understudied, and no imaging biomarker has been identified. Emerging data in recent years suggest an association between the development of chronic systemic inflammation and the development of gray and white matter atrophy and lesions, as well as reduced global and regional cerebral perfusion ^[42–44]^. Decreased white matter volume was found in the cerebrum and the brainstem ^[45,46]^. Existing literature presents inconsistent evidence regarding changes in gray matter volumes in ME/CFS compared with healthy controls ^[45,47,48]^. While an early study reported an overall reduction in gray matter volume ^[49]^, more recent studies found no significant differences ^[47,48]^, and another study observed increased gray matter in the insular cortex and parts of the limbic system ^[45]^. The variability in the findings may reflect the heterogeneous nature of ME/CFS.

Current neuroimaging studies of Long-COVID mainly focus on structural imaging ^[50,51]^. While the structural changes may relate to the Long-COVID pathology, the findings are likely nonspecific, and measurements of such changes rely on ultra-high field imaging facilities ^[52]^. Compared with structural changes, metabolic biomarkers may be more sensitive and helpful to further our understanding of the underlying pathophysiological mechanisms of ME/CFS.

Therefore, the goal of this mini-review is to summarize existing literature on the metabolic neuroimaging findings in ME/CFS and Long-COVID, highlighting their similarities and differences to improve our understanding of these conditions. Specifically, we focus on studies that examine alterations in brain metabolism, such as glucose utilization, oxygen consumption, and key metabolite levels, using techniques like PET and magnetic resonance spectroscopy (MRS). While imaging approaches such as PET-based assessments of neuroinflammation are undoubtedly important ^[53,54]^, they are not included in this review as they do not directly measure metabolic changes. In addition to summarizing current findings, we aim to identify key knowledge gaps and propose directions for future research that may help advance the field and inform the development of diagnostic and therapeutic tools.

## 2. Glucose metabolism

Glucose is the primary source of energy for the human brain and accounts for up to 20% of the body’s total glucose consumption ^[55]^. In healthy individuals, glucose is metabolized via glycolysis to generate adenosine triphosphate (ATP), which is utilized to drive cerebral and cognitive functioning ^[55]^. When glucose metabolism is impaired, due to conditions such as neuroglycopenia, individuals can experience a variety of symptoms, including cognitive and autonomic dysfunction ^[56]^. The similar clinical manifestations between neuroglycopenic patients and patients with ME/CFS and Long-COVID underscore the need to investigate glucose metabolism as a potential pathophysiological mechanism underlying ME/CFS and Long-COVID.

A common method for assessing brain glucose metabolism is [^18^F] fluorodeoxyglucose positron emission tomography (FDG-PET). FDG is a glucose analog in which the hydroxyl group at the 2-position is replaced with the radioactive isotope fluorine-18. This modification prevents FDG from progressing beyond the second step of glycolysis, leading to its intracellular accumulation in metabolically active regions ^[57]^. When paired with PET imaging, FDG enables quantitative and spatially resolved measurements of glucose uptake, offering valuable insights into both global and regional brain metabolism. As such, FDG-PET is an intriguing tool for investigating metabolic dysfunction in ME/CFS and Long-COVID.

### 2.1 FDG-PET in ME/CFS

Early FDG-PET studies identified metabolic abnormalities in ME/CFS. In 1998, Tirelli et al ^[58]^ investigated areas of hypometabolism among right-handed patients with ME/CFS using FDG-PET. The subjects were comprised of 18 patients who fulfilled the CDC case definition of ME/CFS. The study included six age-matched healthy controls screened for physical health, neurologic, and psychiatric diseases. Normalized FDG-PET images of ME/CFS patients revealed significant glucose hypometabolism in the right medial frontal cortex and brainstem compared with healthy controls. Given the medial frontal cortex and brain stem’s respective involvement in autonomic and executive functioning, these findings support a physiological basis for several reported symptoms of ME/CFS, such as fatigue, unrefreshing sleep, and brain fog.

Siessmeier et al ^[59]^ further utilized FDG-PET to investigate areas of cerebral glucose metabolism in patients with ME/CFS. The study included 26 patients with ME/CFS and 18 healthy controls. While 12 ME/CFS patients exhibited no significant metabolic abnormalities, 12 patients showed hypometabolism of the cingulate gyrus and adjacent mesial and cortical regions. Among these 12 patients, five also exhibited further hypometabolism of the orbitofrontal cortex. The remaining two patients demonstrated metabolic deficits in the cuneus and precuneus. The fact that nearly half of the ME/CFS patients did not exhibit glucose hypometabolism highlights the heterogeneous nature of ME/CFS, suggesting that glucose metabolic dysfunction may be present in only a subset of patients and/or that individual differences exist in disease severity, duration, or phenotype that contribute to variations in metabolic abnormalities. These findings also reinforce the presence of region-specific glucose hypometabolism in ME/CFS, which may underlie cognitive dysfunction and functional impairments in affected individuals.

More recently, Sahbai et al ^[^^60]^ evaluated brain hypometabolism, via FDG-PET, and perfusion, via magnetic resonance imaging (MRI), in a 21-year-old woman fulfilling the Fukuda criteria for ME/CFS. Significant hypometabolism was observed in various posterior cortical regions, the amygdalo-hippocampal complexes, and the cerebellum. Significant impairments in short-term memory, visual selective attention, and cognition via neuropsychological testing were consistent with the observed regions of hypometabolism. Notably, no structural abnormalities were detected on MRI, suggesting that metabolic alterations may precede structural changes.

### 2.2 FDG-PET in Long-COVID

Emerging PET studies suggest that Long-COVID shares similar metabolic abnormalities with ME/CFS, particularly in regions associated with cognitive function and autonomic regulation. Verger et al ^[61]^ conducted a retrospective FDG-PET study analyzing brain scans of 143 patients with persistent fatigue, pain, cognitive deficits, insomnia, hyposomnia/anosmia, dysgeusia/ageusia, or signs of dysautonomia three months following COVID-19 infection. This study utilized a previously identified hypometabolic pattern affecting frontal-orbital olfactory regions, limbic/paralimbic areas, the brainstem, and the cerebellum. Hypometabolism was categorized as mild-to-moderate when an incomplete hypometabolic pattern involving two or three of these regions or mild-to-moderate hypometabolism affecting all four regions was observed. Severe hypometabolism was identified when additional brain regions were affected. The results showed that 53% of patients demonstrated normal glucose metabolism, 21% exhibited mild-to-moderate hypometabolism, and 26% displayed severe hypometabolism. Among these hypometabolic individuals, the brainstem and cerebellum were particularly affected. These results further reinforce the notion that persistent metabolic dysfunction may play a role in Long-COVID symptomatology, especially in a subset of patients.

In another retrospective study, Guedj et al ^[62]^ analyzed FDG-PET scans of 35 patients with Long-COVID who had persisting fatigue and functional symptoms, including dyspnea, hyposmia/anosmia, dysgeusia/ageusia, cognitive impairment, insomnia, and pain, for a minimum of 3 weeks following COVID-19 infection. In contrast to 44 healthy controls, patients with Long-COVID demonstrated significant bilateral hypometabolism in the rectal/orbital gyrus, pons, medulla, cerebellum, and the right temporal lobe and right thalamus. Among these patients, a trend emerged suggesting that individuals with more symptoms or who have lived with Long-COVID for a longer duration may exhibit a greater degree of hypometabolism. Although these results are limited by the small sample size, further investigation is warranted to elucidate the progression of the disease over time and how it may result in more substantial metabolic deficits.

A longitudinal study by Horowitz et al ^[63]^ evaluated the persistence of metabolic impairment in patients with Long-COVID over time using whole-brain volume element (voxel)-based analysis of FDG-PET scans. This study included 56 patients with Long-COVID who met the WHO criteria for the condition and 51 healthy controls. Two scans were conducted at averages of 7 and 16 months post-COVID infection. Compared with healthy controls, patients with Long-COVID demonstrated significant glucose hypometabolism in the frontotemporal cortex, medial temporal cortex, insula, pons, and cerebellum. However, among Long-COVID patients, there was no significant change in hypometabolism between the first and second scans, indicating that metabolic dysfunction in Long-COVID remained stable over time, rather than resolving spontaneously.

**Figure 3.**
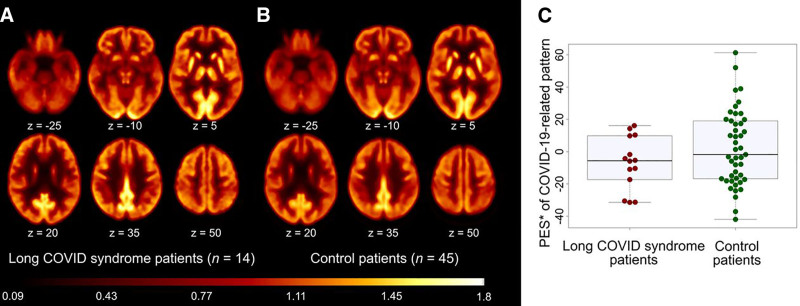
**Group averaged and spatially normalized FDG-PET scans of patients with Long-COVID (A) and healthy control patients (B) ^[65]^.** PES did not differ significantly between patients with Long-COVID and healthy control patients (C) ^[65]^. This research was originally published in *J Nucl Med*, 2022. 63(7): p. 1058-1063. © SNMMI ^[65]^. FDG-PET, fluorodeoxyglucose positron emission tomography; PES, pattern expression score.

In another study, Sakamoto et al ^[64]^ examined regional alterations in glucose metabolism in 22 patients with mild Long-COVID and 20 healthy controls using FDG-PET. Glucose metabolism was conveyed as a *Z*-score, indicating the degree of deviation from the mean. Although no significant differences in glucose metabolism were observed upon initial analysis, the average *Z*-score, normalized by the control group, revealed localized metabolic alterations. Patients exhibited hypometabolism of the left prefrontal medial and lateral areas and bilaterally in the prefrontal medial, sensorimotor, anterior and posterior cingulate, precuneus, super parietal, and mesial temporal areas. Hypermetabolism was observed in the right lateral prefrontal regions and bilaterally in the inferior parietal, mesial, lateral occipital, lateral temporal areas, cerebellum, and pons. Though these findings were not statistically significant, further investigation is warranted into localized neurological alterations in glucose metabolism resulting from Long-COVID. Dressing et al ^[65]^ investigated cerebral glucose metabolism in 14 patients with Long-COVID who presented with persistent neurocognitive symptoms greater than 3 months following COVID-19 infection ^[65]^. FDG-PET images of patients with Long-COVID were compared with 45 healthy controls using a pattern expression score (PES) of a previously derived COVID-19 spatial covariance pattern. PES was established using a spatial pattern rating algorithm to quantify metabolic impairment. Contrary to earlier findings, this study found no significant differences in PES between Long-COVID patients and controls as shown in Figure 3 ^[65]^. Additionally, there was no correlation between metabolic deficits and cognitive impairment, as assessed by the Montreal Cognitive Assessment.

Although much of the current research points to localized regions of hypometabolism resulting from Long-COVID, discrepancies exist. Possible explanations for this inconsistency might be the different analysis pipelines used and the variations in Long-COVID classification, with one study requiring persisting symptoms 3 weeks following infection, while others required several months of ongoing illness. Moving forward, the adoption of standardized diagnostic criteria for Long-COVID will allow comparisons between studies.

## 3. Oxygen metabolism

Oxygen metabolism is essential for sustaining normal neuronal function and preserving brain tissue integrity. Assessing oxygen extraction fraction (OEF) and cerebral metabolic rate of oxygen may be important for understanding both normal brain function and the pathophysiology of neurological disorders. Over the past decades, several oxygen-15 (^15^O) PET imaging techniques have been developed to provide key metabolic measurements, and ^15^O PET is considered the gold standard for in vivo assessment of regional brain oxygenation ^[66–70]^. However, ^15^O PET studies for ME/CFS or Long-COVID are still lacking. Alternatively, advanced MRI techniques, including magnetic resonance spectroscopy (MRS), have emerged as powerful, noninvasive tools for investigating oxygen metabolism in the brain. These techniques provide crucial insights into neurovascular function, metabolic alterations, and potential mechanisms underlying ME/CFS and Long-COVID.

### 3.1 Oxygen extraction fraction

OEF measures the difference in oxygenation between the arteries and veins, and it reflects how much oxygen is extracted from the blood as it passes through the brain. OEF abnormalities may indicate impaired cerebral oxygen utilization, which could contribute to symptoms such as fatigue, cognitive dysfunction, and autonomic dysregulation seen in ME/CFS and Long-COVID.

For example, our group conducted a preliminary study ^[71]^ on Long-COVID patients who met ME/CFS diagnostic criteria (*n* = 8), classic ME/CFS patients (*n* = 9), and healthy controls (*n* = 9) to compare global venous oxygenation through a T2-relaxation-under-spin-tagging MRI and arterial oxygenation using a pulse oximeter. We found significantly elevated OEF levels in the Long-COVID ME/CFS group compared with the other groups. This study provided a direct comparison between the Long-COVID ME/CFS and the classic ME/CFS group, highlighting the differences between the groups.

Another study investigated OEF in neurological post-COVID condition (neuro-PCC), assessing 25 neuro-PCC participants (8 previously hospitalized and 17 nonhospitalized) and 59 age-matched healthy controls ^[72]^. In this study, neuro-PCC was loosely defined as having at least one cognitive or neuropsychiatric symptom after COVID-19 infection, such as brain fog, fatigue, or sleep disturbance. Results revealed significant differences in OEF across the three groups, even after adjusting for age and sex. Post-hoc analysis showed that previously hospitalized neuro-PCC participants had significantly higher OEF than both uninfected controls and nonhospitalized neuro-PCC participants. The study also explored associations between OEF and neurobehavioral measures in neuro-PCC participants ^[72]^. Elevated OEF was significantly associated with impaired locomotor function as assessed by the 4-meter gait speed score (the speed an individual walks 4 meters, measured in meters per second).

While few studies have investigated the OEF in patients with ME/CFS and Long-COVID, these early results highlight the need for further research to better understand the role of altered oxygen utilization in these conditions.

## 4. Lactate

Lactate is a byproduct of anaerobic glycolysis produced when oxygen availability is insufficient for oxidative phosphorylation. Under normal physiological conditions, the brain primarily relies on aerobic metabolism to generate ATP, resulting in minimal lactate accumulation. However, using the MRS technique, several studies by Shungu et al ^[73]^ reported significantly elevated ventricular lactate concentration in patients with ME/CFS compared with healthy volunteers ^[73–76]^. Using magnetic resonance spectroscopic imaging (MRSI), Mueller et al ^[77]^ identified increased lactate-to-creatine ratios in the right insula, thalamus, and cerebellum of ME/CFS patients. This widespread elevation of lactate may reflect a shift toward global anaerobic glycolysis, indicative of impaired energy metabolism.

### 4.1 Other metabolites: insights from MRS

**Figure 4.**
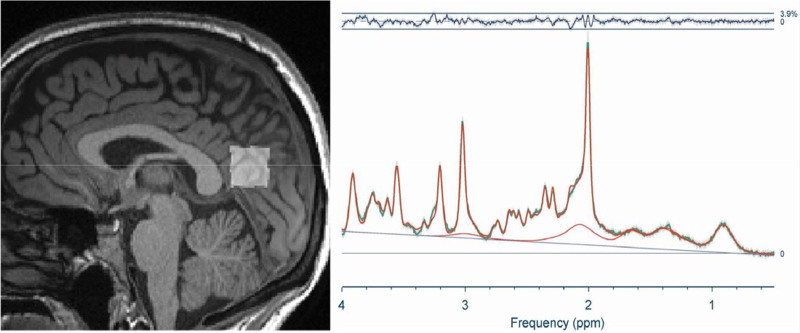
MRI-based MRS scan of the posterior cingulate cortex from Thapaliya et al ^[80]^. The left panel shows the voxel placement, and the right panel shows an average spectrum from the 10 healthy controls. Reproduced from *Am J Med*, 2025. 138(3): p. 567-574.e1. MRI, magnetic resonance imaging; MRS, magnetic resonance spectroscopy.

MRS is a noninvasive tool that provides biochemical insights into brain metabolism ^[78]^. It is most commonly performed on a single voxel of interest ^[79]^, ensuring a higher signal-to-noise ratio and more reliable metabolite quantification. Additionally, its relatively short acquisition time makes it easier to integrate into an MRI session. Figure 4 shows a sample MRS voxel placement and the representative spectra. However, because MRS measurements are limited to one or a few selected regions, assessing whole-brain metabolite levels remains challenging. Moreover, MRS data processing requires specialized expertise, and not all metabolites are quantified with the same level of accuracy ^[81,82]^. Despite these limitations, MRS remains a valuable technique for studying biochemical abnormalities associated with neurological disorders.

## 5. Glutamate/glutamine

Recently, Thapaliya et al ^[80]^ investigated neurochemical alterations in the posterior cingulate cortex, using MRS, across three cohorts: individuals with ME/CFS meeting the Canadian Consensus Criteria or International Consensus Criteria (*n* = 17); individuals with Long-COVID, defined by symptom onset within 3 months following SARS-CoV-2 infection and persisting for at least three months per the WHO working case definition (*n* = 17); and healthy controls with no history of chronic illness or prior COVID-19 infection (*n* = 10) ^[80]^. They reported significantly elevated glutamate (Glu) levels in the posterior cingulate cortex of both ME/CFS and Long-COVID groups compared with healthy controls, but no significant difference between the two patient groups. Glutamate (Glu) is an essential excitatory neurotransmitter in the brain. Elevated Glu levels in both patient groups may be attributed to the accumulation of inflammatory cytokines and activation of glial cells. Proinflammatory cytokines, such as interleukin-1β, interleukin-6, and tumor necrosis factor, have been reported to be increased in Long-COVID and ME/CFS ^[83]^. These cytokines affect the release, transportation, and metabolism of Glu, leading to increased extracellular Glu concentration in the brain.

This same study also attempted to measure glutamine (Gln) levels. However, since the MRS peaks (chemical shifts) of Gln and Glu are very close at clinical MRI field strength, typically Gln cannot be measured without spectral editing techniques. The combined signal Glx (glutamine+glutamate) is typically quantified ^[84]^. Compared with healthy controls, Glx levels were increased in ME/CFS patients but not in Long-COVID patients. The Glx levels were found to be negatively correlated with physical function in Long-COVID patients. In contrast, Glx levels were positively correlated with physical function in ME/CFS patients. This inconsistency in association could be due to the small sample size used in the study or reflect a true difference between the two conditions, which remains to be elucidated further.

## 6. γ-aminobutyric acid

As a primary inhibitory neurotransmitter, γ-aminobutyric acid (GABA) plays a crucial role in maintaining neural homeostasis. Marinkovic et al ^[85]^ were the first to report significantly reduced cortical GABA levels in the occipital cortex of individuals with Long-COVID, suggesting a state of cortical hyperexcitability. Taken together, symptoms of ME/CFS and Long-COVID may indicate an altered excitation–inhibition balance. Excess Glx could drive Glu-mediated excitotoxicity, oxidative stress, and mitochondrial dysfunction, while insufficient GABAergic inhibition may exacerbate neuroinflammation, further disrupting cognitive processing, attentional control, and executive function. These neurochemical imbalances may contribute to the cognitive deficits commonly described as “brain fog” and mental fatigue in affected individuals.

## 7. *N*-acetylaspartate

*N*-acetylaspartate (NAA) is synthesized in neuronal mitochondria and plays a crucial role in metabolic processes that support cell signaling. Since NAA generates the most prominent peak in MRS scans of healthy individuals ^[86]^, it is routinely used as a key marker in spectroscopy studies. NAA levels reliably capture shifts in neuronal metabolic homeostasis, with reduced NAA levels consistently reported in conditions such as traumatic brain injury ^[86,87]^, neuroinflammation ^[88]^, and other neurological disorders ^[89]^, all of which disrupt normal metabolism. In a small study, Brooks et al ^[83]^ reported significantly reduced NAA levels in the hippocampus of ME/CFS patients (*n* = 7) compared with healthy controls (*n* = 10). The lower NAA levels observed in people with ME/CFS may indicate neuronal injury or dysfunction in the hippocampus.

Marinkovic et al ^[85]^ conducted a single-voxel MRS study in the occipital cortex in 18 patients (24.4 ± 5.2 years) who fulfilled the WHO diagnostic criteria for Long-COVID ^[85]^. They found that NAA levels were lower in the Long-COVID group compared with a healthy control group, though the decrease was not significant. The NAA levels were inversely associated with scores on a brief test of recent verbal memory in the Long-COVID group. Conversely, the study by Thapaliya et al ^[80]^ found elevated NAA levels in the posterior cingulate cortex of Long-COVID patients compared with healthy controls. A plausible interpretation is that this elevation may reflect a compensatory response to reduced brain metabolic efficiency, where increased glucose and oxygen utilization lead to elevated NAA production ^[90]^.

## 8. Choline

Choline (CHO) is essential for acetylcholine production and is a key component of the cell membrane. In MRS, CHO levels are valuable for detecting abnormalities in cell membrane turnover ^[91]^. Leveraging the MRSI technique, Mueller et al ^[77]^ reported elevated CHO levels in the ME/CFS group in the bilateral anterior cingulate cortex (ACC), left middle cingulate, right calcarine sulcus, and right occipital and temporal lobes. Other studies found increased CHO in the occipital cortex ^[92]^, frontal white matter ^[93]^, and basal ganglia ^[94]^. Since MRS detects only free CHO and water-soluble membrane breakdown products (eg, phosphocholine and glycerophosphocholine), elevated CHO is often interpreted as evidence of abnormal phospholipid metabolism and increased cell membrane turnover, consistent with glial proliferation and neuroinflammatory processes.

## 9. Glutathione

ME/CFS has been associated with increased oxidative stress. Glutathione (GSH) is a crucial antioxidant that protects cells from oxidative stress by neutralizing reactive oxygen species ^[95]^ and maintaining cellular redox balance ^[96]^. Using MRS, Shungu et al ^[73]^ first examined occipital lobe cortical GSH levels in ME/CFS patients compared with healthy volunteers. They found significantly reduced GSH levels in ME/CFS patients. Additionally, GSH levels were found to be inversely correlated with ventricular lactate levels and were significantly associated with various indices of physical health and disability in ME/CFS patients. In a subsequent study, Godlewska et al ^[97]^ investigated GSH levels in the ACC of ME/CFS patients versus healthy controls. They found significantly lower GSH levels in the rostral portions of the ACC, particularly in the pregenual ACC. These findings suggest that reduced cortical GSH, indicative of redox imbalance and neuroinflammation, may play a key role in the pathophysiology of ME/CFS ^[98]^.

There is currently no study of GSH in Long-COVID within the existing literature. However, several relevant studies have examined GSH levels in COVID-19 survivors ^[99,100]^. For instance, Poletti et al ^[100]^ used MRS to investigate GSH levels in COVID-19 survivors after 3 to 12 months of hospital admissions, finding reduced GSH levels in the ACC. This study also confirmed persistent systemic inflammation in postacute COVID-19.

## 10. Discussion

Advanced imaging techniques such as FDG-PET and MRS have enabled the detection of metabolic dysfunctions in ME/CFS and Long-COVID that cannot be captured by conventional structural imaging. This review summarized the existing literature on metabolic neuroimaging in both conditions, with a focus on glucose metabolism, oxygen utilization, and other key metabolites. The metabolites discussed in this review are summarized in Table 1.

**Table 1:** Summary of the metabolic and neurochemical findings across ME/CFS and Long-COVID.

Condition	Glucose	Oxygen	Lactate	Glu	Gln	GABA	NAA	CHO	GSH
ME/CFS	Regional decreases: Right medial frontal cortex and brainstem ^[58]^ Cingulate gyrus and adjacent mesial and cortical regions, orbitofrontal cortex, cuneus, and precuneus ^[59]^ Posterior cortical regions, amygdalo-hippocampal complexes, and cerebellum ^[60]^	-	Global elevation ^[73–77]^:	Regional elevation: posterior cingulate cortex ^[80]^	Regional elevation: posterior cingulate cortex ^[80]^	-	Regional decrease: hippocampus ^[83]^	Regional elevations: Bilateral ACC, left middle cingulate, right calcarine sulcus, and right occipital and temporal lobes ^[77]^ Frontal white matter ^[89]^ Basal ganglia ^[90]^	Regional decreases: Occipital lobe ^[73]^ ACC ^[93]^
Long-COVID	Regional decreases: Brainstem ^[61]^, medulla ^[62]^, and pons ^[62,63]^ Cerebellum ^[61–63]^ Rectal/orbital gyrus, right temporal lobe, and right thalamus ^[62]^ Frontotemporal cortex, medial temporal cortex, and insula ^[63]^	Global elevation: Long-COVID patients that fulfill ME/CFS criteria ^[71]^	-	Regional elevation: posterior cingulate cortex ^[80]^	-	Regional decrease: Occipital cortex ^[85]^	Regional decrease: occipital cortex ^[85]^ Regional elevation: posterior cingulate cortex ^[80]^	-	-

Diagonal slashes indicate knowledge gaps in the current literature; ACC, anterior cingulate cortex; CHO, Choline; Gln, glutamine; Glu, glutamate; GSH, Glutathione; ME/CFS, Myalgic Encephalomyelitis/Chronic Fatigue Syndrome; NAA, *N*-acetylaspartate.

An emerging body of neuroimaging research has provided valuable insights into the metabolic alterations underlying ME/CFS and Long-COVID, revealing both commonalities and distinctions between these conditions. Evidence across multiple studies suggests that both disorders exhibit glucose hypometabolism, particularly in brain regions associated with cognitive function, autonomic regulation, and fatigue, such as the frontal cortex, cingulate gyrus, brainstem, and cerebellum. Neurotransmitter imbalances, particularly elevated Glu levels, have also been reported in both conditions and may reflect neuroinflammation and glial activation ^[86]^. In addition, reduced GSH levels, an indicator of oxidative stress, have been observed in ME/CFS and in post-COVID-19 survivors, suggesting redox imbalance may be a shared pathophysiological feature.

Despite these similarities, elevated oxygen extraction has been observed in Long-COVID and neuro-PCC compared with controls, but not to classic ME/CFS. Additionally, increased brain lactate levels have been reported in ME/CFS, while corresponding data in Long-COVID remain absent. Together, these findings may point to a compensatory mechanism in Long-COVID, when the brain is extracting more oxygen to maintain metabolic hemostasis, whereas in ME/CFS, mitochondrial dysfunction leads to a shift toward anaerobic glycolysis.

At the same time, several conflicting findings highlight the complexity and heterogeneity of these disorders. While glucose hypometabolism is widely reported in Long-COVID, some studies have failed to detect significant metabolic differences from healthy controls, suggesting possible variation in disease severity, duration, or methodological differences in PET imaging. Similarly, the NAA levels in Long-COVID remain inconsistent, with some studies reporting reduced levels, while others found elevation. In contrast, ME/CFS studies consistently report reduced NAA levels, particularly in the hippocampus. Another key discrepancy lies in the Glx findings, where ME/CFS patients showed significantly increased Glx levels compared with controls, while in Long-COVID, Glx levels did not significantly differ from controls but correlated negatively with physical function, which is a trend opposite to that observed in ME/CFS. A GABA deficit, observed in one Long-COVID study, suggests potential cortical hyperexcitability, though no comparable data exists for ME/CFS.

Although both conditions share hallmark symptoms such as fatigue, cognitive impairment, and postexertional malaise, neuroimaging studies suggest their metabolic underpinning may differ. ME/CFS is a heterogeneous disorder with diverse disease triggers and a prolonged disease course, often developing years after an initial viral or immune insult. In contrast, Long-COVID presents a more temporally defined postviral syndrome with onset directly following SARS-CoV-2 infection. Long-COVID patients also tend to be younger, more medication-naive, and have shorter disease duration compared with ME/CFS. It is therefore reasonable to hypothesize that Long-COVID patients, especially those who fulfill the diagnostic criteria for ME/CFS, represent a relatively homogeneous subset. Investigating their metabolic abnormalities offers a unique opportunity to investigate the mechanisms underpinning postviral fatigue syndromes.

Only few studies to date have directly compared Long-COVID and ME/CFS in parallel. Comparative studies are critical for identifying both shared and distinct metabolic signatures, which may illuminate unique pathophysiological mechanisms and guide targeted interventions. As can be seen from above, the current studies reviewed involve varied definitions of Long-COVID; some follow the WHO case definition, while earlier studies were conducted in loosely defined COVID survivors. As standard diagnostic criteria become available, further studies should use the WHO diagnostic criteria or a well-accepted ME/CFS case definition. This will enhance comparability across studies and improve meta-analytic integration. Current literature also reflects considerable variation in imaging analysis methods. For example, among FDG-PET studies, Horowitz et al ^[63]^ employed whole-brain voxel-based analysis, Dressing et al ^[65]^ used a PES from a predefined spatial covariance pattern, and Verger et al ^[61]^ relied on visual rating. It is also important to acknowledge that neuroimaging approaches, such as FDG-PET and advanced MRI, require specialized, costly equipment and extensive technical expertise, which limit their routine clinical implementation. Consequently, most of the studies reviewed are limited by small sample sizes, which calls for caution when interpreting findings and reinforces the importance of replication in larger, well-powered cohorts. Additionally, important confounding factors, such as medication use, comorbid conditions, and illness duration, are often not systematically reported or controlled for, further limiting the generalizability of the findings. Future studies should strive to account for these variables to better isolate disease-specific metabolic changes. We recommend that future studies adopt harmonized analysis pipelines and, where possible, share de-identified imaging data in public repositories. This will facilitate pooled analyses and retrospective comparisons, accelerating progress in understanding these complex conditions.

In terms of research directions, more targeted research into underexplored metabolic domains is needed. More research in oxygen metabolism in ME/CFS and Long-COVID could be valuable since dyspnea and dysfunctional breathing are prevalent symptoms in both conditions, but few studies so far have been conducted. In addition, GSH, GABA, and CHO metabolism in Long-COVID remain poorly characterized and warrant further investigation. Additional studies focused on these aspects may uncover important mechanistic pathways and identify biomarkers of disease severity or progression. Finally, longitudinal studies are essential to understand how metabolic changes evolve over time and whether they persist, resolve, or predict clinical outcomes. This approach may help distinguish between transient postviral effects and long-term pathophysiological alterations and inform the development of early diagnostic or prognostic tools.

Beyond diagnosis and pathophysiological investigation, metabolic neuroimaging holds promise as a tool for monitoring treatment response in ME/CFS and Long-COVID. A small case study by Mairal et al ^[101]^ demonstrated the potential utility of FDG-PET in tracking the therapeutic effects of hyperbaric oxygen therapy in a 31-year-old ME/CFS patient. Pretreatment FDG-PET imaging revealed hypometabolism in key brain regions commonly affected in ME/CFS, including the amygdala, hippocampus, thalamus, and cerebellum, when compared with age-matched controls. Post-treatment imaging suggested metabolic normalization in these regions, coinciding with clinical improvement, highlighting FDG-PET’s potential to detect brain changes associated with symptom modulation. Similarly, MRS has shown value in evaluating biochemical response to targeted interventions. In a pilot study using MRS, Weiduschat et al ^[102]^ investigated the effects of supplementing a GSH precursor, *N*-acetylcysteine (NAC), on cortical antioxidant status in ME/CFS patients ^[102]^. After 4 weeks of daily NAC intake, cortical GSH levels in ME/CFS patients increased significantly, normalizing to levels comparable to healthy controls. Together, these findings suggest that metabolic neuroimaging can offer objective, noninvasive markers of treatment response.

## 11. Conclusions

Metabolic neuroimaging has revealed important insights into the pathophysiology of ME/CFS and Long-COVID, identifying shared features such as glucose hypometabolism and elevated Glu, as well as distinct features in oxygen extraction. However, despite these advances, substantial gaps in knowledge remain. Many metabolic pathways, such as lactate, CHO, and GSH in Long-COVID, remain insufficiently studied. To advance the field, future research should adopt standardized diagnostic criteria, implement harmonized acquisition protocols and analysis pipelines, promote data sharing, and include longitudinal studies. Early findings suggest that metabolic imaging holds potential as a biomarker for treatment response, warranting its incorporation in larger, controlled clinical trials. Continued investment in imaging research is essential to close current knowledge gaps and to develop reliable diagnostic, mechanistic, and therapeutic tools for these complex and often debilitating conditions.

## Conflicts of interest

The authors have no conflicts of interest to declare.

## Funding

This work is supported by NIH grants R21NS129120, R01NS136806, and R01NS117638.

## Acknowledgements

We thank Ms. Sera Saju and Michelle Blate for their support and assistance.
